# Hypoxia upregulates expression of human endosialin gene via hypoxia-inducible factor 2

**DOI:** 10.1038/sj.bjc.6604685

**Published:** 2008-09-23

**Authors:** A Ohradanova, K Gradin, M Barathova, M Zatovicova, T Holotnakova, J Kopacek, S Parkkila, L Poellinger, S Pastorekova, J Pastorek

**Affiliations:** 1 Centre of Molecular Medicine, Institute of Virology, Slovak Academy of Sciences, Dubravska cesta 9, Bratislava 845 05, Slovak Republic; 2 Department of Cell and Molecular Biology, Karolinska Institutet, Stockholm 17177, Sweden; 3 Institute of Medical Technology and School of Medicine, University of Tampere, Biokatu 6, FIN-33014, Finland

**Keywords:** endosialin, HIF-2, Ets-1, hypoxia, angiogenesis

## Abstract

Endosialin is a transmembrane glycoprotein selectively expressed in blood vessels and stromal fibroblasts of various human tumours. It has been functionally implicated in angiogenesis, but the factors that control its expression have remained unclear. As insufficient delivery of oxygen is a driving force of angiogenesis in growing tumours, we investigated whether hypoxia regulates endosialin expression. Here, we demonstrate that endosialin gene transcription is induced by hypoxia predominantly through a mechanism involving hypoxia-inducible factor-2 (HIF-2) cooperating with the Ets-1 transcription factor. We show that HIF-2 activates the endosialin promoter both directly, through binding to a hypoxia-response element adjacent to an Ets-binding site in the distal part of the upstream regulatory region, and indirectly, through Ets-1 and its two cognate elements in the proximal promoter. Our data also suggest that the SP1 transcription factor mediates responsiveness of the endosialin promoter to high cell density. These findings elucidate important aspects of endosialin gene regulation and provide a rational frame for future investigations towards better understanding of its biological significance.

Endosialin (alternatively named TEM1 or CD248) is a highly glycosylated single-pass transmembrane protein proposed to become a marker of tumour vasculature and a target for anticancer therapy (Teicher, 2007). Endosialin was discovered with a monoclonal antibody (MAb) raised against human foetal fibroblasts as an antigen selectively expressed in tumour blood vessels and absent in normal human tissues (Rettig *et al*, 1992). Independently, TEM1 (tumour endothelial marker 1) was linked to tumour endothelium by serial analysis of gene expression (St Croix *et al*, 2000). Identity of endosialin with TEM1 was then demonstrated by Christian *et al* (2001), who cloned its full-length cDNA, and based on the domain composition and homology to thrombomodulin classified endosialin as a C-type lectin-like receptor.

Subsequent studies have confirmed that endosialin is upregulated in blood vessels and activated stromal fibroblasts in human colorectal, brain and breast tumours as well as in mouse xenograft models (Carson-Walter *et al*, 2001; Brady *et al*, 2004; Davies *et al*, 2004; Madden *et al*, 2004; Dolznig *et al*, 2005; MacFadyen *et al*, 2005, 2007; Huber *et al*, 2006; Rupp *et al*, 2006). Endosialin expression is restricted to capillaries and shows a heterogeneous pattern typical for molecules involved in vascular reorganisation. Moreover, abundant endosialin expression was found in the vasculature and fibroblast-like cells of the developing mouse and human embryos (Rupp *et al*, 2006; MacFadyen *et al*, 2007; Virgintino *et al*, 2007). Using a gene targeting approach, Nanda *et al* (2006) have shown that endosialin is dispensable for normal development and subcutaneous tumour growth, but can modulate invasiveness and metastatic progression in an orthotopic xenograft model of colorectal cancer. All these data support the functional involvement of endosialin in angiogenesis, a complex process of vascular branching and sprouting that plays a key role in tumour expansion and progression, and therefore represents an opportunity for therapeutic intervention against cancer.

Recent antiangiogenic strategies are based on targeting the molecules selectively expressed in tumour vessels (Fukumura and Jain, 2007). From this viewpoint, expression pattern and possible functional implication in angiogenesis make endosialin a promising subject of clinical investigations. However, present research suffers from inadequate knowledge of factors and pathways that control endosialin expression and determine its tissue distribution. There is only a single study showing that mouse endosialin gene transcription is induced by high cell density, but the underlying molecular mechanism has not been elucidated (Opavsky *et al*, 2001).

Angiogenesis is well known to be principally driven by hypoxia in growing solid tumours as well as in developing embryos (Liao and Johnson, 2007). Hypoxia often develops in intratumoral areas with deficient or functionally inadequate blood vessels. Insufficient delivery of oxygen then promotes multiple adaptive responses including angiogenesis, which facilitates new blood supply and allows for further tumour expansion (Harris, 2002). This adaptation process is governed by hypoxia-inducible transcription factors, notably HIF-1 and HIF-2. In normoxia, the *α*-subunits of HIFs are modified by oxygen-dependent prolyl hydroxylases (PHDs), interact with the von Hippel-Lindau tumour-suppressor protein, and undergo rapid ubiquitination and proteasome-mediated degradation. Hypoxia leads to inactivation of PHDs, stabilisation of the *α*-subunits, their translocation into the nucleus, heterodimerisation with the constitutive *β*-subunit, and formation of an active transcription complex (Ruas and Poellinger, 2005). Hypoxia-inducible factors can transactivate a large spectrum of genes contributing to adaptation, including proangiogenic factors VEGF (vascular endothelial growth factor), ANG2 (angiopoietin 2) and their receptors (Hickey and Simon, 2006; Ruas *et al*, 2007).

Here, we provide the first evidence that hypoxia induces expression of endosialin predominantly through the HIF-2 transcription factor. We have identified *cis*-elements in the upstream regulatory region of the human endosialin gene that mediate the hypoxic responses and demonstrate that these elements bind HIF-2 in both direct and indirect manners. Moreover, we show that upregulation of endosialin by high cell density is mediated by the SP1 transcription factor and is partly caused by pericellular hypoxia. Thus, these combined transcriptional regulatory mechanisms are important for intratumoral expression of endosialin.

## Materials and methods

### Cell culture

All cell lines, including human HeLa cervical carcinoma cells, 42-MG-BA glioblastoma cells (Perzelová *et al*, 1998), FIB-3 placental fibroblasts, mouse NIH3T3 fibroblasts, b.END3 brain endothelial cells and the HIF-1*β*-deficient Hepa-1c4 hepatoma cells (Legraverend *et al*, 1982) were cultured in DMEM with 10% FCS (BioWhittaker, Verviers, Belgium) in a humidified atmosphere with 5% CO_2_ at 37°C. The cells were exposed to hypoxia (2% O_2_) in an anaerobic workstation (Ruskinn Technology, Bridgend, UK) in 5% CO_2_, 2% H_2_ and 91% N_2_ at 37°C. Hypoxia was also induced chemically with 100 *μ*M 2,2′-dipyridyl (DIP, Sigma, St Louis, MO, USA).

### Reporter promoter constructs

The endosialin-luc promoter construct was generated by an insertion of a −1091/+43 endosialin genomic region amplified by PCR upstream of the firefly luciferase gene in pGL3-basic luciferase reporter vector (Promega, Madison, WI, USA). Deletion and mutant variants of the promoter were generated from this construct by PCR using the primers mentioned in the Supplementary information. Deletion variant −285/+43 was created by *Sac*I digestion.

### Expression plasmids

Human HIF-1*α* and mouse HIF-2*α* cDNAs in pcDNA1/Neo plasmid were kindly provided by Professor Patrick Maxwell, Imperial College of Science, Technology and Medicine, London, UK. Expression plasmid pCMV-HIF-1*β* containing human HIF-1*β*/ARNT cDNA was described previously (Whitelaw *et al*, 1993). Expression plasmids CMV-Sp1 containing human SP1 cDNA and SP(RSV)AP-2 containing human TFAP2A/AP-2 cDNA were purchased from Addgene (Cambridge, MA, USA). pcDNA3.1/Neo/Ets-1 expression plasmid was created by insertion of 1571 bp human Ets-1 cDNA fragment amplified by RT–PCR into pcDNA3.1(+) (Invitrogen, Carlsbad, CA, USA). Human endosialin cDNA was amplified using endosialin_F1 and endosialin_R1 primers and cloned into phCMV1 expression vector (Genlantis, San Diego, CA, USA). For prokaryotic expression, the endosialin cDNA fragment encoding the amino acids 192–675 was amplified and cloned into pGEX-4T-1 plasmid (Amersham Biosciences, Chalfont St Giles, UK).

### Generation of endosialin-specific MAb

BALB/c mice were immunised with two doses of 5 × 10^6^ HeLa cells transfected with endosialin cDNA in a phCMV1 expression vector and boosted with 100 *μ*g GST-endosialin protein bound to Glutathione Sepharose 4B. Fusion of spleen cells with Sp2/0 myeloma cells was carried out 3 days later. Monoclonal antibodies produced by the hybridomas were screened for the specific reactivity towards endosialin protein in HeLa-endosialin *vs* HeLa-mock cells by ELISA and immunoblotting. The hybridoma culture (M78) was subcloned by limiting dilution, expanded and used for the MAb production.

### RNA isolation and reverse transcription PCR

Total RNA was extracted from cells using the NucleoSpin RNA II kit (Macherey-Nagel, Düren, Germany) and reverse transcribed with the M-MuLV reverse transcriptase (Finnzymes, Espoo, Finland) using random heptanucleotides as primers. Polymerase chain reactions were performed using the primers for endosialin (endosialin_F2, R2), VEGF-A, *β*-actin and DyNAmo HS SYBR Green qPCR Kit (Finnzymes). Following an initial denaturation at 95°C for 10 min, the amplification programme was set to denaturation at 95°C for 30 s, annealing at 60°C for 40 s and extension at 72°C during 40 s for a total of 40 cycles, and finally 5 min at 72°C.

### Transient transfection and luciferase assay

The cells were plated into 35 mm Petri dishes to reach approximately 70% monolayer density on the next day. Transfection was performed with 2 *μ*g of pGL3-based promoter construct and 100 ng of pRL-TK plasmid DNAs using a GenePorterII reagent (Genlantis). Alternatively, 1 *μ*g of pGL3-based promoter construct, 1 *μ*g of expression vector containing the transcription factor's cDNA and 50 ng of pRL-TK plasmids were transfected. One day later, the transfected cells were trypsinised and plated in triplicates into 24-well plates. The cells were allowed to attach overnight and then transferred to hypoxia for additional 24 h. Reporter gene expression was assessed using the Dual-Luciferase Reporter Assay System (Promega) and the luciferase activity was normalised against the renilla activity.

### Immunoblotting

FIB-3 and 42-MG-BA cells were plated in sparse (12 000 cells cm^−2^) or dense (48 000 cells cm^−2^) cultures, left to attach overnight and then incubated for 24 h in normoxia and hypoxia, respectively. Proteins were extracted with cold RIPA buffer (1% Triton X-100, 0.1% sodium deoxycholate and 1x Complete protease inhibitor cocktail (Roche, Mannheim, Germany) in PBS) for 15 min at 4°C, and total protein concentrations were determined by BCA assay (Pierce, Rockford, IL, USA). Samples of 50–80 *μ*g were separated by SDS–PAGE and blotted onto the PVDF membrane. The membrane was treated with M78 MAb in undiluted hybridoma medium followed by the peroxidase-conjugated goat anti-mouse immunoglobulins (DAKO, Glostrup, Denmark) and developed using enhanced chemiluminescence. For loading control, the membrane was probed with anti-*α*-tubulin antibody (rat monoclonal YL1/2, Novus Biologicals, Littleton, CO, USA) and the polyclonal goat anti-rat IgG-HRP (Santa Cruz Biotechnology, Santa Cruz, CA, USA).

### RNA interference

RNA duplexes targeting HIF-1*α* (Hs_HIF1A_5 HP Validated siRNA), HIF-2*α* (Hs_EPAS1_5 HP Validated siRNA) and negative control siRNA were purchased from Qiagen (Qiagen AB, Solna, Sweden). 42-MG-BA cells were transfected with 10 nM siRNA using HiPerFect reagent (Qiagen) according to the manufacturer's instructions. After 24 h transfection, the medium was changed and cells were transferred to hypoxia for additional 24 h. Then, proteins were extracted with cold high-salt buffer (50 mM Tris-HCl pH 7.4, 500 mM NaCl, 1% NP-40 and 20% glycerol) supplemented with 0.5 mM PMSF and 5 mM 2-mercaptoethanol for 30 min at 4°C. Lysates were cleared by centrifugation for 30 min at 16 000 **g** and subjected to immunoblotting analysis as described above.

### Chromatin immunoprecipitation assay

FIB-3 cells were plated into 500 cm^2^ plates at the density of 12 000 cells cm^−2^, left to attach overnight and incubated in absence or presence of 100 *μ*M DIP for 4 h and 24 h. The cells were fixed in 1% formaldehyde in PBS for 10 min at room temperature. Chromatin isolation, shearing to a size of ∼600–800 bp and its immunoprecipitation with antibodies against HIF-1*α* (rabbit polyclonal, Santa Cruz Biotechnology), HIF-1*β* (rabbit polyclonal, Mason *et al*, 1994) and HIF-2*α* (rabbit polyclonal, Novus Biologicals), was performed as described in Löfstedt *et al*, 2004. As a negative control and for chromatin preclearing, anti-human IgG antibodies (rabbit polyclonal, Abcam, Cambridge, UK) were used. Purified DNA was subjected to 36 cycles of PCR with primers flanking the putative hypoxia-responsive elements (HREs) (−1072HRE, −976HRE, −574HRE) and Ets-binding sites (−171/−133-EBS) within the endosialin promoter. Primers flanking the HRE of the VEGF promoter were used as a positive control.

### Bioinformatics

*In silico* analysis of the endosialin promoter was performed using rVISTA 2.0 (Loots and Ovcharenko, 2004) and MatInspector (Quandt *et al*, 1995) programs.

## Results

### Hypoxia induces expression of endosialin

**Figure 1:**
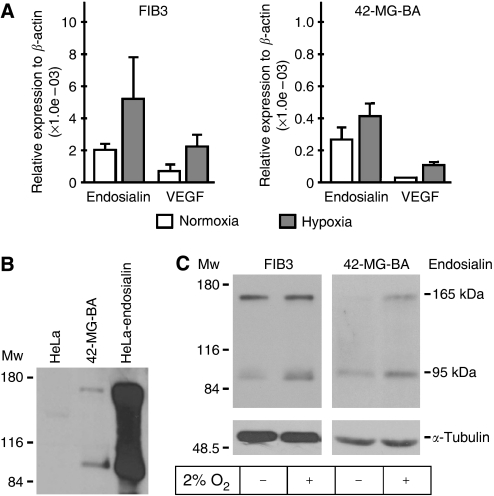
Expression of endosialin in response to hypoxia. (**A**) FIB-3 and 42-MG-BA cells were incubated for 24 h in normoxic or hypoxic conditions. Total RNA was extracted and expression of endosialin mRNA was analysed by quantitative RT–PCR and normalized to *β*-actin levels. Vascular endothelial growth factor (VEGF) mRNA was used as a positive control. (**B**) Specificity of the monoclonal antibody (MAb) M78 for endosialin protein was evaluated by immunoblotting using 42-MG-BA cells that naturally express endosialin and HeLa cells transfected with endosialin cDNA (HeLa-endosialin). Mock-transfected HeLa cells served as a negative control. M78 MAb could recognise both the non-glycosylated core protein (95 kDa) and the highly glycosylated endosialin (165 kDa). (**C**) Immunoblotting analysis of endosialin protein levels in FIB-3 (50 *μ*g total proteins/lane) and 42-MG-BA cells (80 *μ*g total proteins/lane loaded due to lower endosialin expression level, see part a). The cells were incubated for 24 h in hypoxia (2% oxygen, +) or normoxia (−). M78 MAb revealed increased expression of endosialin in the hypoxic cells. *α*-Tubulin antibody was used for a loading control.

In initial experiments we examined the effect of hypoxia on expression of endosialin in two human cell lines, namely FIB-3 placental fibroblasts and 42-MG-BA glioblastoma cells. The cells were exposed to hypoxia (2% O_2_) for 24 h, and parallel samples were incubated for the same time period in normoxia. In both cell lines, the endosialin gene showed increased expression in hypoxic conditions as demonstrated by qRT–PCR (Figure 1A). We then verified the hypoxic induction of endosialin at the protein level using our specific MAb M78 generated against HeLa cells transfected with the endosialin cDNA. The M78 MAb could recognise a ∼95 kDa core protein as well as a 165 kDa glycosylated form of endosialin (Figure 1B) and detected higher endosialin level (especially that of the core protein) in cells exposed to hypoxia than in normoxic cells (Figure 1C). These initial experiments represented a proof-of-concept and prompted us to analyse the transcriptional regulation of endosialin.

### HIF-2 transcription factor mediates hypoxic induction of endosialin

Computer analysis of a 2 kb fragment of the 5′ flanking region of the human endosialin gene revealed several putative HRE with the core sequence 5′-RCGTG-3′, both in sense and antisense orientations. Out of these, three HREs at positions −1072/−1065, −976/−969 and −574/−567 (numbered with respect to the transcription initiation site) were predicted as the conserved binding sites for the transcription factors of the HIF family.

**Figure 2:**
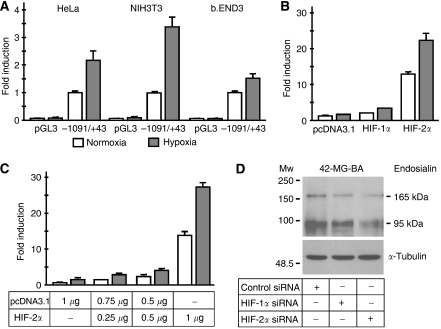
Effects of hypoxia, hypoxia-inducible factor (HIF)-1*α* and HIF-2*α* on the activity of endosialin promoter. (**A**) HeLa, NIH 3T3 and b.END3 cells were transfected with the −1091/+43 promoter fragment in pGL3-basic luciferase vector (2 *μ*g) and pRL-TK plasmid (100 ng). The transfected cells were incubated for 24 h in normoxia or hypoxia. Luciferase activity was normalised against renilla activity. Hypoxic values were expressed as a fold induction of the normoxic ones. Empty pGL3-basic plasmid was used for control of basal luciferase activity. Similar hypoxic induction was observed in all three cell lines. (**B**) HeLa cells were co-transfected with the −1091/+43 pGL3-basic promoter construct (1 *μ*g), pRL-TK plasmid (50 ng), and HIF-1*α* or HIF-2*α* expression vectors (1 *μ*g each). Empty pcDNA3.1 was used to adjust total DNA content in the control sample. The transfected cells were treated in normoxia and hypoxia and the promoter activity was assessed as described above. HIF-2*α* but not HIF-1*α* dramatically induced endosialin promoter activity. (**C**) HeLa cells were co-transfected with constant amounts of the −1091/+43 pGL3-basic promoter construct (1 *μ*g) and pRL-TK plasmid (50 ng), and an increasing amount of HIF-2*α* expression vector. Total DNA was adjusted with empty pcDNA3.1. Endosialin promoter activities were assessed as above and showed a dose-dependent induction by HIF-2*α*. The induction was more prominent in hypoxia apparently due to both stabilisation and activation of HIF-2*α* protein. (**D**) Immunoblotting analysis of endosialin protein level in the 42-MG-BA cells transfected with the specific HIF-1*α*, HIF-2*α* and control siRNAs and incubated under hypoxia for 24 h. Both forms of endosialin were decreased after treatment with HIF-1*α* and HIF-2*α* siRNAs, respectively, but the effect of HIF-2*α* siRNA was more evident suggesting a predominant role of HIF-2 in control of endosialin expression.

Therefore, we cloned the −1091/+43 region of the human endosialin gene into the promoter-less pGL3-basic vector and analysed its activity in a dual luciferase reporter assay. Initial attempts to perform the promoter analyses in endosialin-positive human cell lines were hampered by low survival of these cells following transfection. For this reason, we decided to use the endosialin-negative HeLa cells that are easy to transfect and have been frequently used in studies of HIF-mediated signalling. To prove that these cells enable proper transcriptional responses of the endosialin promoter, we included for comparison two mouse cell lines, namely NIH3T3 fibroblasts and b.END3 endothelial cells that naturally express the endosialin gene. In all cases, hypoxic treatment of the transfected cells led to an increased expression of the firefly luciferase gene driven by the endosialin upstream regulatory region when compared to the normoxic controls (Figure 2A). The level of hypoxic response in HeLa cells was similar to that observed in NIH3T3 and b.END3 cells. Thus, the subsequent promoter experiments were mostly carried out using this human cell line.

To test whether HIF family members are responsible for the hypoxic activation of endosialin gene, we first analysed the activity of the endosialin promoter in Hepa-1c4 cells deficient in HIF-1*β* (ARNT), a common heterodimerisation partner for HIF-1*α* and HIF-2*α*. As expected, hypoxic induction of the endosialin promoter was abrogated in the absence of functional HIF-1*β*, but was restored when the −1091/+43 promoter construct was co-transfected with HIF-1*β* expression vector (data not shown). We then analysed the response of the endosialin promoter to HIF-1 and HIF-2 transcription factors. HeLa cells were co-transfected with the −1091/+43 promoter construct together with HIF-1*α* and HIF-2*α* expression vectors, respectively, or with an empty expression vector, and cultivated in normoxia or hypoxia for 24 h. Co-transfection with HIF-1*α* resulted in modest effects on endosialin promoter activity. In contrast, expression of HIF-2*α* led to robust, dose-dependent activation of the endosialin promoter both in normoxia and in hypoxia demonstrating that HIF-2*α* has strong functional activity on the endosialin promoter (Figure 2B and C).

These findings were further supported by RNA interference-mediated knock-down of endogenous HIF-1*α* and HIF-2*α*, respectively. Hypoxic 42-MG-BA glioblastoma cells transfected with the specific siRNAs clearly showed reduced endosialin protein level when compared to the cells transfected with the control siRNA (Figure 2D). Moreover, the effect of HIF-2*α* knock-down was more dramatic than that of HIF-1*α*, in line with the proposed predominant role of HIF-2*α* in endosialin regulation under hypoxia.

### HIF-2 regulates the endosialin promoter by binding to HRE −976/−969

**Figure 3:**
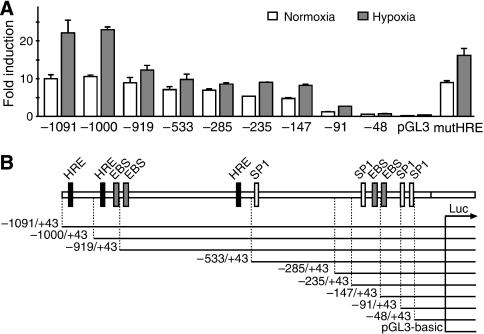
Localisation of hypoxia-responsive regions in the endosialin promoter. (**A**) HeLa cells were transfected with 5′-progressively truncated endosialin promoter fragments in pGL3-basic luciferase vector (2 *μ*g) and pRL-TK plasmid (100 ng). MutHRE construct contained mutations in two HREs (−1072/−1065 and −976/−969). The transfected cells were incubated for 24 h in normoxia or hypoxia. Luciferase activity was normalised against renilla activity. Empty pGL3-basic plasmid was used for control of the basal luciferase activity. (**B**) Scheme of the upstream regulatory region of the endosialin gene with indicated positions of predicted binding sites for transcription factors of the HIF family (HRE), ETS family (EBS) and SP1. Truncated promoter fragments are illustrated below with the 5′ termini scaled to scheme to facilitate allocation of the relevant *cis* elements.

To identify regulatory elements that are responsible for the hypoxia-dependent activation, we constructed a series of 5′-deletion variants of the originally cloned endosialin promoter region as well as a variant with a double-point mutation in the putative HREs −1072/−1065 and −976/−969 (designated as mutHRE). Luciferase activity driven by these promoter variants was tested in HeLa cells incubated for 24 h in hypoxia and normoxia, respectively. As shown in Figure 3, maximal hypoxic induction was detected with the promoter constructs containing an intact −1091/−919 region, indicating that HRE −1072/−1065 and/or HRE −976/−969 might participate in the HIF-mediated response.

**Figure 4:**
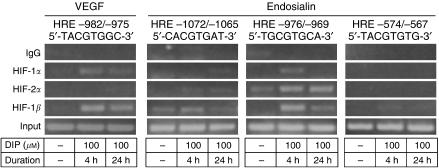
The binding of hypoxia-inducible factor (HIF) subunits to hypoxia-responsive elements in the endosialin promoter. Chromatin immunoprecipitation assays using FIB-3 cells treated with 100 *μ*M DIP for 4 and 24 h, respectively. Untreated cells were used as a negative control. The cells were incubated with formaldehyde for crosslinking, DNA was extracted and immunoprecipitated with antibodies specific for HIF subunits or for human IgG antibodies to visualise background signal. Recovered DNA was used in PCR with primers flanking respective HREs. DNA samples from non-immunoprecipitated extracts were amplified as input. HIF-2*α* was found to bind to HRE −976/−969, but not to the other HREs.

We next performed chromatin immunoprecipitation assays, allowing for the analysis of transcription factors binding to their cognate sequences in an innate context of the promoter. We used FIB-3 cells, which naturally express the endosialin gene as well as both the HIF-1*α* and HIF-2*α* subunits (data not shown). The cells were treated for 4 and 24 h, respectively, with 100 *μ*M DIP (a cell-permeable iron chelator commonly used in the studies of hypoxic responses due to inhibition of prolyl and asparaginyl hydroxylases) to induce stable and transcriptionally active HIF proteins. After treatment, the cells were fixed with formaldehyde, their chromatin was isolated, sheared and immunoprecipitated with antibodies directed to all three subunits of HIF-1 and HIF-2 transcription factors. Precipitated DNA fragments were purified and used in PCRs together with primers flanking the putative HRE −1072/−1065 and HRE −976/−969 sites, as well as the HRE −574/−567 site. Primers flanking the HRE −982/−975 in the VEGF promoter were used for a positive control. We found that only the endosialin HRE −976/−969 showed a consistent signal for HIF-1*α*, HIF-2*α* and HIF-1*β* in DIP-treated cells when compared to untreated control cells (Figure 4). In a manner similar to the co-transfection experiments, the signals resulting from immunoprecipitation with the anti-HIF-2*α* antibody were stronger than those with the anti-HIF-1*α* antibody, suggesting that HIF-2 may be a dominant factor in regulation of the endosialin promoter.

### HIF-2 cooperates with Ets-1

**Figure 5:**
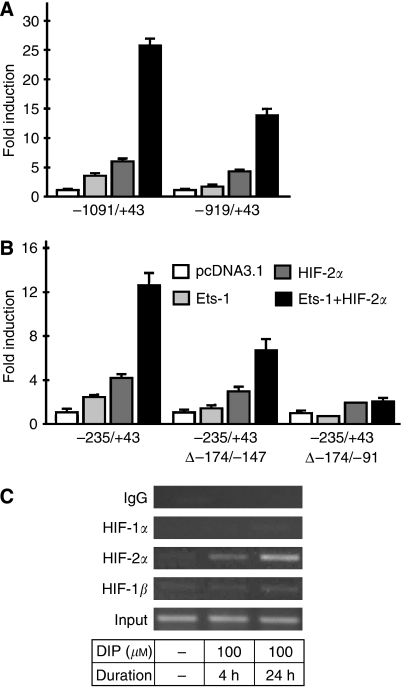
Cooperation between the hypoxia-inducible factor (HIF)-2 and Ets-1 transcription factor. (**A**) HeLa cells were co-transfected with the −1091/+43 or −919/+43 pGL3-basic promoter construct (1 *μ*g), pRL-TK plasmid (50 ng) and HIF-2*α* and/or Ets-1 expression vectors (1 *μ*g each). Empty pcDNA3.1 was used to adjust total DNA content in all samples to 3 *μ*g. The transfected cells were incubated in hypoxia and the promoter activity was assessed as described in Figure 2. Simultaneous expression of HIF-2*α* and Ets-1 showed synergic effect on endosialin promoter activity. (**B**) HeLa cells were transfected with the −235/+43 pGL3-basic promoter construct and its mutated variants with deletions of one (Δ−174/−147) or both (Δ−174/−91) EBS elements, respectively, and pRL-TK, HIF-2*α* and/or Ets-1 plasmids were co-transfected as above. The graph shows the promoter activity obtained in hypoxic cells only, as a similar profile was observed in the normoxic counterparts. (**C**) Chromatin immunoprecipitation assay was performed on FIB-3 cells under same conditions as described in Figure 4, except that primers flanking the ETS transcription factor-binding site (EBS) elements were used to detect binding of the HIF subunits. HIF-2*α* clearly bound to the EBS-containing region.

Recently it was reported that a small group of genes, regulated by HIF-2 rather than HIF-1, have at least one HRE in proximity to an ETS transcription factor-binding site (EBS) and that HIF-2*α* and ETS-family member physically interact and cooperate in regulation of these genes (Aprelikova *et al*, 2006). Consistent with this concept, two putative EBS elements were found in the endosialin promoter less than 80 bp downstream of HRE −976/−969 at positions −935/−919 and −907/−891. Moreover, co-transfection of the −1091/+43 endosialin promoter construct with an expression vector encoding a representative member of ETS family, Ets-1, resulted in augmented endosialin promoter activity indicating that Ets-1, in fact, contributes to regulation of endosialin gene expression (Figure 5A).

To assess possible cooperation between Ets-1 and HIF-2 in regulation of endosialin gene expression, we co-transfected HeLa cells with the −1091/+43 promoter construct and Ets-1 and HIF-2*α* expression vectors either separately or together. Noteworthy, the combined activating effect of Ets-1 and HIF-2*α* was about 2.5 times higher than that calculated for an additive effect of these two transcription factors expressed individually. This fact suggests that Ets-1 and HIF-2*α* act synergistically to transactivate endosialin transcription (Figure 5A). Furthermore, the −919/+43 promoter fragment lacking EBS −935/−919 displayed reduced activation of the endosialin promoter by Ets-1 in the presence of HIF-2*α* (Figure 5A), whereas no further reduction was observed when EBS −907/−891 was deleted (not shown). These data strongly suggest that hypoxic induction of endosialin gene is governed by HIF-2 transcription factor cooperating with Ets-1 by adjacent HRE −976/−969 and EBS −935/−919 elements.

Nonetheless, the existence of the functional HRE nearly 1 kb upstream of the transcription start site could not explain our observation that shorter promoter constructs lacking the classical HRE sequence still remained partly hypoxia-responsive (Figure 3). Moreover, in the absence of any apparent HRE, these constructs responded to co-transfection with the HIF-2*α* expression vector by increased reporter gene activity (Figure 5B). Interestingly, a similar situation was described for the core promoter of VE-cadherin gene that was transactivated by HIF-2*α* cooperating with Ets-1 by two EBS elements (Le Bras *et al*, 2007). Indeed, the endosialin promoter was found to contain two additional EBS motifs: the first one designated as Ets-1 or ELF-2-binding site (EBS −171/−156), the other one as GABP-binding site (EBS −149/−133). To investigate whether any of these putative sites is functional, we deleted either EBS −171/−156 alone or both EBS elements from the −235/+43 fragment and co-transfected HeLa cells with the resulting promoter constructs in the absence or presence of Ets-1 and HIF-2*α* vectors. As shown in Figure 5B, induction of −235/+43 reporter gene activity caused by simultaneous as well as individual expression of Ets-1 and HIF-2*α* was considerably decreased following the deletion of EBS −171/−156 and was almost completely abolished when both EBS elements were eliminated.

Moreover, we detected the physical presence of the HIF-2 transcription factor on the −235/−16 endosialin promoter region by chromatin immunoprecipitation assays (Figure 5C). On the basis of these results, we propose that hypoxic induction of the endosialin promoter fragment lacking apparent HREs is mediated by HIF-2 that acts in cooperation with the Ets-1 transcription factor binding to both EBS elements.

### SP1 activates endosialin promoter in dense cells

**Figure 6:**
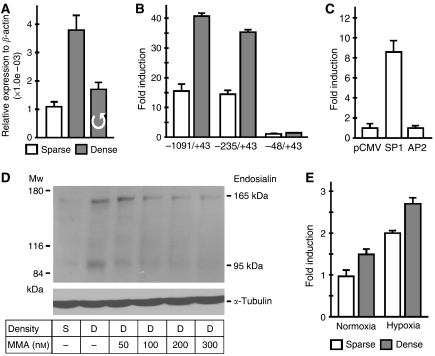
Expression of endosialin in response to high cell density. (**A**) 42-MG-BA cells were plated to form sparse (S, 12 000 cells cm^−2^) and dense (D, 48 000 cells cm^−2^) monolayers and incubated for 24 h in normoxia. Gentle stirring was applied to dense cells to eliminate pericellular hypoxia. Total RNA was extracted and expression of endosialin mRNA was analysed by quantitative RT–PCR and normalised to *β*-actin levels. Stirring reduced the density-elevated expression of endosialin. (**B**) Endosialin promoter activity in sparse *vs* dense HeLa cells was assessed by transfection of −1091/+43, −235/+43 and −48/+43 fragments in pGL3-basic vector (1 *μ*g) with pRL-TK plasmid (50 ng) as described in Figure 2. Effect of density was not observed in the shortest promoter construct. (**C**) Sparse HeLa cells were co-transfected with −235/+43 promoter fragment in pGL3-basic (1 *μ*g), pRL-TK plasmid (50 ng) and SP1 or AP-2 expression vectors (1 *μ*g each). Empty pCMV plasmid was used to adjust total DNA content in the control sample. The promoter activity was assessed after 24 h incubation in normoxia as described in Figure 2. SP1 but not AP-2 induced endosialin promoter activity. (**D**) Immunoblotting analysis of endosialin expression in sparse (S) and dense (D) 42-MG-BA cells treated with increasing concentrations of the SP1 inhibitor mithramycin A (MMA). M78 MAb revealed increased expression of endosialin in the dense cells and progressively reduced levels in MMA-treated cells. *α*-Tubulin antibody was used for a loading control. (**E**) Additive effect of high cell density and hypoxia on endosialin promoter activity was evaluated in HeLa cells transfected with the −1091/+43 promoter fragment as described in Figure 2A.

Given that high cell density increases the expression of the mouse endosialin gene (Opavsky *et al*, 2001), we analysed the effect of cell crowding on expression levels of the human endosialin gene by qRT–PCR. As shown in Figure 6A, increased cell density led to elevated expression of endosialin mRNA. Interestingly, endosialin expression was reduced in cells subjected to gentle stirring throughout the experiment to ensure proper oxygenation of the cell monolayer and elimination of pericellular hypoxia, which occurs in high density cultures (Sheta *et al*, 2001). This finding indicates that the effect of cell density on endosialin expression is partially mediated by pericellular hypoxia. To obtain further insight into a potential mechanism of transcriptional control of the human endosialin gene by cell density, we analysed the endosialin promoter activity in HeLa cells grown in sparse *vs* dense monolayers. We found that the −1091/+43 and −235/+43 promoter fragments showed more than two-fold higher activity in dense cells when compared to the sparse culture (Figure 6B). Conversely, the activity of the −48/+43 promoter construct was not modulated by cell density, suggesting that the density-responsive regulatory region is localised upstream of the nucleotide −48.

Computer analysis of the sequence between nucleotides −235 and −48 relative to the transcriptional start site revealed that this region is GC-rich and possesses several SP1- and AP-2-binding sites. Co-transfection of the −235/+43 promoter construct with either SP1 or AP-2 expression vectors clearly demonstrated that SP1 (and not AP-2) plays a role in activation of the endosialin promoter (Figure 6C). This conclusion was supported by immunoblotting analysis, which showed that density-induced induction of endosialin protein expression in 42-MG-BA cells can be abrogated by the SP1 inhibitor mithramycin A (Figure 6D).

Interestingly, the effect of cell density on endosialin gene expression could be observed both under normoxic and hypoxic conditions. In hypoxia, cell density further augmented the hypoxic induction, indicating that these regulatory mechanisms may be integrated in modulation of endosialin expression (Figure 6E).

## Discussion

Several studies have reported selective upregulation of endosialin in the vasculature and activated fibroblasts of various human tumours, and a link between endosialin expression and induced angiogenesis has been proposed. However, the molecular mechanisms that control endosialin expression have remained elusive (Rettig *et al*, 1992; Brady *et al*, 2004; Davies *et al*, 2004; Madden *et al*, 2004; Dolznig *et al*, 2005; MacFadyen *et al*, 2005; Huber *et al*, 2006).

In this study, we demonstrate for the first time that hypoxia, which is a driving force of angiogenesis under both physiological and pathological conditions, regulates endosialin gene transcription and that the hypoxic induction is mediated predominantly by the HIF-2 transcription factor. Our finding is compatible with the expression pattern and target gene preference of the HIF-2*α* subunit. In contrast to the structurally and functionally related HIF-1*α* subunit that is ubiquitously expressed, HIF-2*α* is confined to tissues and cell types, which also express endosialin, including blood vessels, fibroblasts and glial cells (Wiesener *et al*, 2003). In addition, HIF-2*α* (rather than HIF-1*α*) regulates a subset of genes involved in angiogenic responses, such as Tie-2/TEK, VEGFR1 (Flt-1), VEGFR2 (Kdr/Flk-1), VE-cadherin, EPO and TGF-*α* (Tian *et al*, 1997; Takeda *et al*, 2004; Elvert *et al*, 2003; Raval *et al*, 2005; Le Bras *et al*, 2007). Moreover, the kinetics of endosialin induction by hypoxia with a maximum level achieved during a 24 h incubation period as well as an optimal oxygen concentration between 2 and 5% (data not shown) better correspond to the temporal profile and activation optimum of HIF-2*α*, which increases with time at moderate-to-mild hypoxia, whereas HIF-1*α* stabilisation occurs at lower oxygen concentration and its levels are reduced at prolonged hypoxia (Löfstedt *et al*, 2007). Thus, the endosialin appears to be a new angiogenesis-related target of the HIF-2 transcription factor.

In further support of this model, the Ets-1 transcription factor contributes to activation of endosialin expression. Physical and functional interaction between HIF-2 and ETS family members appears to be required for the proper hypoxic regulation of several HIF-2-target genes (Aprelikova *et al*, 2006). Here, we demonstrate that HIF-2 cooperating with Ets-1 can transactivate the endosialin promoter through two distinct regions: a distal region, possibly serving as an enhancer and a proximal region representing the core promoter. The distal region contains a module composed of the functional HRE site and the adjacent Ets-1-binding site. In such a constellation, HIF-2 can directly bind DNA and at the same time interact with Ets-1 through the N-terminal transactivation domain, as has been shown for the other HIF-2-target genes including VEGFR2 (Elvert *et al*, 2003). The proximal region of the endosialin promoter is hypoxia-responsive despite the absence of any apparent HRE. On this part of the promoter, HIF-2 seems to operate through the interaction with Ets-1 and its two cognate EBS elements. The paradigm for this mode of HIF-2 action can be found in VE-cadherin promoter (Le Bras *et al*, 2007).

The proximal promoter region mediates also a cell density-dependent activation response. This effect is partly related to pericellular hypoxia and as such might again involve HIF-2, the stabilisation and activation of which requires just a mild lowering of oxygen tension (Löfstedt *et al*, 2007). As the SP1 transcription factor is capable to cause a considerable induction of endosialin in sparse monolayer cell culture, and inhibition of SP1 reduces induction of endosialin expression by high cell density, SP1 seems to be a critical factor mediating this regulatory effect.

**Figure 7:**
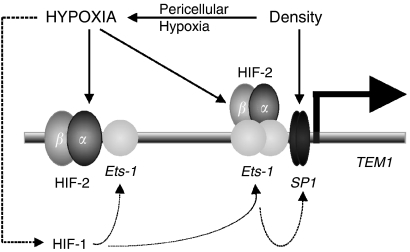
Schematic illustration of the molecular anatomy of the endosialin promoter and regulatory pathways induced by hypoxia and cell density. Distal part of the promoter contains adjacent hypoxia-responsive element (HRE) and ETS transcription factor-binding site (EBS) element and transmits hypoxic signalling through hypoxia-inducible factor (HIF)-2 directly binding to DNA and cooperating with Ets-1. Proximal part of the promoter contains two EBS elements (but not HRE) and hypoxia signalling is mediated by HIF-2 through Ets-1 and two EBS elements. High cell density contributes to endosialin activation by means of SP1-binding sites in the proximal promoter and partially feeds the hypoxic pathway by pericellular hypoxia. Moreover, hypoxia might influence activation of endosialin indirectly through HIF-1 that upregulates expression of Ets-1, which in turn upregulates expression of SP1. On the basis of this model, hypoxia and density cross-talk to induce endosialin transcription.

Thus, as schematically summarised in Figure 7, the transcription factors HIF-2 and Ets-1 mediate hypoxic responses, whereas SP1 functions as a mediator of density effects. Importantly, there is a cross-talk between these regulatory pathways. However, the situation might be more complex, as SP1 was found to be a transcriptional target of Ets-1 (Sato and Furukawa, 2007), and Ets-1 expression is enhanced by HIF-1 (Oikawa *et al*, 2001). In the case of endosialin expression, we observed that the gene is regulated by hypoxia under conditions that stabilise and activate HIF-2 and/or HIF-1. It is conceivable that HIF-1 does not need to be directly bound to endosialin promoter to mediate this effect, but induces Ets-1 expression and thus supports formation of the cooperative complex between Ets-1 and HIF-2. Elevated expression of Ets-1 then presumably leads to increased transcription of SP1, which can further stimulate activity of endosialin promoter.

The involvement of hypoxia in regulation of endosialin gene expression sheds new light on its possible biological significance in vascular remodelling. According to recent data, endosialin is preferentially associated with pericytes and stromal fibroblasts, but not with endothelial cells, as suggested in earlier studies (MacFadyen *et al*, 2005; Christian *et al*, 2008; Simonavicius *et al*, 2008). Pericytes form an outer sheath around the endothelium and play an important role in maturation of blood microvessels and stabilisation of vascular network, whereas stromal fibroblasts stimulate angiogenesis by trophic effects and extracellular matrix reorganisation (Jain, 2003; Armulik *et al*, 2005). In contrast to normal tissues, tumours are characterised by continuously ongoing angiogenesis with high proliferation rate of endothelial cells and diminished maturation of blood vessels (Bergers and Benjamin, 2003). The functionally defective vasculature gives rise to hypoxia in the tumour tissue, resulting in excessive production of proangiogenic factors and a new round of angiogenesis accompanied by tumour expansion. To allow for a new blood vessel formation, the pericytes of pre-existing vessels have to be detached from endothelial cells and enable them to respond to mitogenic signals. It is possible that hypoxia (and also dense intercellular contacts) upregulate expression of endosialin to relieve pericyte–endothelial interactions and/or to modulate local tumour stroma, and thereby facilitate migration of dividing endothelial cells and sprouting of new blood vessels. Indeed, endosialin was found to support an invasive tumour phenotype in the mouse knock-out model and modulate *in vitro* migration, which are phenomena well known to be linked to hypoxia and angiogenesis (Nanda *et al*, 2006; Tomkowicz *et al*, 2007). This hypothesis could also explain why endosialin expression is detectable in the developing embryo, where new blood vessels form to feed extensive hypoxic areas, but not in adult tissues, where hypoxia is generally absent, and vascular network is usually mature and quiescent.

Although further functional studies are clearly required to prove the above-proposed role for endosialin, this study clarifies important aspects of its regulation and provides a rational frame for future investigations towards a better understanding of its biological significance.
