# Supplementary material for: Hypoxia upregulates expression of human endosialin gene via hypoxia-inducible factor 2
Source: Br J Cancer. 2008 Sep 23;99(8):1348–56. doi: 10.1038/sj.bjc.6604685 (PMC2570523; doi:10.1038/sj.bjc.6604685)
Supplement: Supplementary Table [file 6604685x1.doc]

**Supplementary Table:**

Primers and oligonucleotides used for the cloning and PCR analyses.

| Primer designation (F - forward,   R –reverse) | Sequence  (5’→ 3’) | Purpose |
| --- | --- | --- |
| -1091/+43_F | GGGCAGGGAGGGCTCCACACA | Cloning of the endosialin-luc promoter construct |
| -1091/+43_R | ctcgAGGCCAGCAACAGGCGCAGC |
| pGL3-basic_R | TAGCACGCGTAAGAGCTCGGTA | Cloning of truncated variants of the promoter |
| -1000/+43_F | GTCCCCTCACAGCCTTCCAC |
| -919/+43_F | CAGGAGCCCAGAGAGGCATT |
| -533/+43_F | ACGGTGAGGTGAAGGGACAAA |
| -235/+43_F | CCTGGGGCAGCCGTCAACT |
| -147/+43_F | CCGGGGAAGAGGGAGCAG |
| -91/+43_F | TGAGCCCGGCCCCAGGCC |
| -48/+43_F | CCCAGGCCCTCCCCCTGT |
| -1072/-1065_mut_F | GCTCCACACAC**T**TGATGCCTGT | Cloning of the mutHRE variant |
| -1072/-1065_mut_R | ACAGGCATCA**A**GTGTGTGGAGC |
| -976/-969_mut_F | CACATCCTGC**T**TGCAGCCCAGC |
| -976/-969_mut_R | GCTGGGCTGCA**A**GCAGGATGTG |
| pGL3-basic_F1 | cgctctccatcaaaacaaaacg |
| pGL3-basic_R1 | cgaaatgcccatactgttgagcaattcac |
| -174_R | GGTGGGGGGCTCTAAGGTTG | Deletion of EBSs in the -235/+43 reporter construct |
| -147/+43_F | CCGGGGAAGAGGGAGCAG |
| -91/+43_F | TGAGCCCGGCCCCAGGCC |
| Ets-1_F | AAGTGCCAACTTCCCCTGGAGT | Ets-1 cDNA |
| Ets-1_R | TGTCCTTGGAAGGTCTCAGCAG |
| Endosialin_F1 | cgacttaacagatctcgagctcaagcttcgaattcatgCTGCTGCGCCTGTTGC | endosialin  cDNA |
| Endosialin_R1 | cccgggcccgcggtaccgtcgactgcagaattcttaCACGCTGGTTCTGCAGGTCT |
| Endosialin_F2 | cctgggggaggctggtcttg | Quantitative  RT PCR |
| Endosialin_R2 | cacgctggttctgcaggtctg |
| VEGF-A_F | CAGCACGGTCCCTCTTGGAA |
| VEGF-A_R | CCTCCTCTTCCCTGTCAGGA |
| β-actin_F | CCAACCGCGAGAAGATGACC |
| β-actin_R | AGGATCTTCATGAGGTAGTCAGTC |
| -1072HRE_F | TCCTGGATGATGGGCTCAGTG | Detection of transcription factor binding sites |
| -1072HRE_R | GCAGGATGTGGAAGGCTGTGA |
| -976HRE_F | CACACACGTGATGCCTGTGTCA |
| -976HRE_R | GAGGCAGCCCCTAAACAGGAAAT |
| -574HRE_F | TGACCAAGCTGTTTCCATTCAGG |
| -574HRE_R | GCACTCTCTGCCAAGCTGTTTGT |
| -171/-133-EBS_F | AGAGCTCGGCAGGCAGGCTT |
| -171/-133-EBS_R | TGGCGGCAGCTCTTGACAGG |
| VEGF-HRE_F | GACGTTCCTTAGTGCTGGCGGGTAGGTTTGA |
| VEGF-HRE_R | GGCACCAAGTTTGTGGAGCTGAGAACGGG |

Underlined sequences indicate core motifs with mutated nucleotides in bold, lowercase sequences represent nucleotides inserted for the cloning purposes.
